# Radiomics nomogram based on CT radiomics features and clinical factors for prediction of Ki-67 expression and prognosis in clear cell renal cell carcinoma: a two-center study

**DOI:** 10.1186/s40644-024-00744-1

**Published:** 2024-08-06

**Authors:** Ben Li, Jie Zhu, Yanmei Wang, Yuchao Xu, Zhaisong Gao, Hailei Shi, Pei Nie, Ju Zhang, Yuan Zhuang, Zhenguang Wang, Guangjie Yang

**Affiliations:** 1 Department of Nuclear Medicine, The Affiliated Hospital of Qingdao University, No. 59, Haier Road, Qingdao, 266061 Shandong China; 2 School of Basic Medicine, Qingdao University, Qingdao, Shandong China; 3 Department of Scientific Research Management and Foreign Affairs, The Affiliated Hospital of Qingdao University, Qingdao, Shandong China; 4 GE Healthcare China, Pudong New Town, Shanghai, China; 5 School of Nuclear Science and Technology, University of South China, Hengyang, Hunan China; 6 Department of Pathology, The Affiliated Hospital of Qingdao University, No. 16, Jiangsu Road, Qingdao, 266003 Shandong China; 7 Department of Radiology, The Affiliated Hospital of Qingdao University, No. 16, Jiangsu Road, Qingdao, 266003 Shandong China

**Keywords:** Clear cell renal cell carcinoma, Radiomics, Heterogeneity, Ki-67, Outcome

## Abstract

**Objectives:**

To develop and validate a radiomics nomogram combining radiomics features and clinical factors for preoperative evaluation of Ki-67 expression status and prognostic prediction in clear cell renal cell carcinoma (ccRCC).

**Methods:**

Two medical centers of 185 ccRCC patients were included, and each of them formed a training group (*n* = 130) and a validation group (*n* = 55). The independent predictor of Ki-67 expression status was identified by univariate and multivariate regression, and radiomics features were extracted from the preoperative CT images. The maximum relevance minimum redundancy (mRMR) and the least absolute shrinkage and selection operator algorithm (LASSO) were used to identify the radiomics features that were most relevant for high Ki-67 expression. Subsequently, clinical model, radiomics signature (RS), and radiomics nomogram were established. The performance for prediction of Ki-67 expression status was validated using area under curve (AUC), calibration curve, Delong test, decision curve analysis (DCA). Prognostic prediction was assessed by survival curve and concordance index (C-index).

**Results:**

Tumour size was the only independent predictor of Ki-67 expression status. Five radiomics features were finally identified to construct the RS (AUC: training group, 0.821; validation group, 0.799). The radiomics nomogram achieved a higher AUC (training group, 0.841; validation group, 0.814) and clinical net benefit. Besides, the radiomics nomogram provided a highest C-index (training group, 0.841; validation group, 0.820) in predicting prognosis for ccRCC patients.

**Conclusions:**

The radiomics nomogram can accurately predict the Ki-67 expression status and exhibit a great capacity for prognostic prediction in patients with ccRCC and may provide value for tailoring personalized treatment strategies and facilitating comprehensive clinical monitoring for ccRCC patients.

**Supplementary Information:**

The online version contains supplementary material available at 10.1186/s40644-024-00744-1.

## Introduction

Renal cell carcinoma (RCC) is a prevalent form of cancer worldwide, ranking as the tenth and thirteenth most frequent cancer in men and women, respectively [1]. Clear cell renal cell carcinoma (ccRCC), the most predominant subtype of RCC, accounts for 75-90% of kidney cancers [2, 3]. Despite surgical intervention remaining the cornerstone of management, the 5-year relative survival rate of ccRCC patients remains dismal [4]. Besides, a steady stream of immunotherapeutic drugs has been approved and used for treatment over the past decade, however, a substantial proportion of cases does not demonstrate objective and durable responses when treated with such novel modalities [5–7]. This heterogeneity highlights the imperative role that robust predictive factors play in patient stratification and subsequent individualization of care plans. Therefore, the identification of prognostic biomarkers assumes paramount importance in the stratification of ccRCC patients, aiding clinical decision-making for treatment.

The Ki-67 nucleoprotein, existing in all phases of the cell cycle except resting phase (G0), is closely associated with the status of cellular proliferation [8]. Utilizing standard immunohistochemistry, the Ki-67 index is measured after biopsy or surgery, offering quantitative insights into the heterogeneity and aggressiveness of neoplasms [9]. The Ki-67 index has proved to be a valuable prognostic predictive tool in various malignancies [10–12]. As for ccRCC, previous studies have shown that a high Ki-67 proliferation status (Ki-67 ≥ 15%) serves as an independent prognostic factor, which is strongly correlated with a poor prognosis [13, 14]. For ccRCC patients, particularly those classified as high-risk based on their Ki-67 index, close postoperative monitoring, adjunctive immunotherapy and targeted therapy are recommended [15, 16]. However, the current method of immunohistochemical evaluation from surgical or biopsy specimens is invasive, posing risks such as needle tract implantation and limited reflection of neoplastic heterogeneity [17]. There is an urgent need for a non-invasive, precise, and effective preoperative assessment of the Ki-67 index to overcome sampling biases in clinical practice.

Radiomics, as an emerging technique in oncology, presents a promising approach for the transformation of medical images into quantitative and high-dimensional image features. Through the application of model-building algorithms, radiomics features have the capacity to reveal associations between tumour imaging and histopathology as well as heterogeneity [18–21]. Besides, radiomics has been successfully applied in various oncology fields, such as to distinguish between benign renal masses and RCCs and to predict prognosis in ccRCC [22, 23]. Moreover, it has been utilized to predict the Ki-67 expression status in breast cancer [24], sinonasal malignancies [25], and gastrointestinal stromal tumours [26]. However, to date, radiomics has not been used for predicting Ki-67 expression status in ccRCC.

The purpose of this study was to establish and validate a radiomics nomogram combining radiomics features and clinical factors for preoperative evaluation of Ki-67 expression status and prognostic prediction in ccRCC.

## Materials and methods

### Patients

The study adhered to the principles of the Declaration of Helsinki. The approval was obtained from the Institutional Review Board of the two participating hospitals with the informed consent waived.

This retrospective research involved patients from two hospitals (Shandong Provincial Hospital Affiliated to Shandong First Medical University and the Affiliated Hospital of Qingdao University) between 2015 and 2019. The inclusion criteria were as follows: (1) Histopathological confirmation of ccRCC through surgical procedures, (2) Performance of a CT scan within a 14-day period before the initiation of therapy, (3) Measurement of the Ki-67 index via an immunohistochemical examination following the surgical procedure. Patients with incomplete clinical data or poor image quality were excluded, resulting in the enrollment of 185 patients (121 males and 64 females) with a median age of 58 years (age range: 28 to 87 years).

According to the TRIPOD statement, 130 patients from the Affiliated Hospital of Qingdao University assigned to training group, and 55 patients from Shandong Provincial Hospital Affiliated to Shandong First Medical University assigned to validation group.

**Fig. 1:**
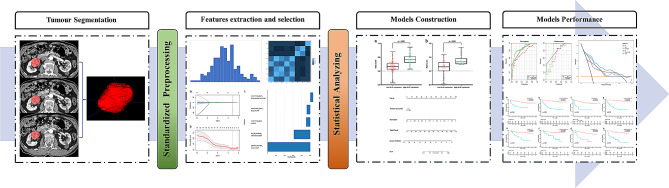
Workflow of the radiomics procedure

The radiomics procedure employed in this research is illustrated in Fig. 1.

### Acquisition of CT images

A volume of 90–100 mL of iodinated contrast medium, with concentrations of 350 mg I/mL or 370 mg I/mL, was intravenously administered via hand or peripheral veins in the elbow using a power injector at a controlled rate of 2.5 - 3.0 mL/s. Subsequently, two post-contrast CT scans were acquired during the corticomedullary phase (CMP) at 30 s and the nephrographic phase (NP) at 90 s after injection. Table 1 presents the CT scan protocols utilized by the 2 participating hospitals.

**Table 1:** CT scan protocols

CT scanner	CT 256	CT 128	CT 64	CT 16	CT 64	CT 16	CT 16
Scanner model	Brilliance iCT 256	Somatom Definition Flash	Somatom Sensation 64	Brilliance 16	Discovery 750	Aquilion One	Brightspeed 16
Manufacturer	Philips	Siemens	Siemens	Philips	General Electric	Toshiba	General Electric
Gantry rotation time (s)	0.5	0.28	0.5	0.5	0.5	0.5	0.6
Tube voltage (kV)	120	120	120	120	120	120	120
Tube current	250 mA	Ref. 200 mAs (Care Dose 4D)	200 mAs	200 mAs	200–400 mA (Automatic tube current modulation)	100–400 mA	100–400 mA
Detector collimation (mm)	0.625	0.6	0.6	0.75	0.625	1	0.625
Matrix	512 × 512	512 × 512	512 × 512	512 × 512	512 × 512	512 × 512	512 × 512
Pitch	0.915	1.0	1.0	1	1.375	0.9375	1.375
Slice thickness (mm)	5	5	5	5	5	5	5
Slice spacing (mm)	5	5	5	5	5	5	5
Reconstruction kernel	B	B30f	B30f	B	standard	FC18	standard
Hospital	a	a	a	a	b	b	b

Note: s (second); kV (kilovolt); mA (milliampere); mm (millimeter); (a) The Affiliated Hospital of Qingdao University; (b) Shandong Provincial Hospital Affiliated to Shandong First Medical University

### Clinical and pathological data collection

The clinical and pathological information were collected, including the age, gender, Ki-67 index, hematuria, lumbago, Eastern Cooperative Oncology Group Performance Status (ECOG-PS), hemoglobin, leukocyte count, platelet count (PLT), lactate dehydrogenase (LDH), alkaline phosphatase (ALP), calcium level, creatinine level, blood urea nitrogen (BUN), tumour size and necrosis (measured and evaluated on CT images). Following surgery, the Ki-67 index was determined using standard immunohistochemistry, which involved calculating the fraction of Ki-67-positive cells. According to the previous study [13], the ccRCC patients in this investigation were stratified into two groups based on their Ki-67 expression status, categorized as either high (≥ 15%) or low (<15%) Ki-67 expression.

### Analysis of the clinical information and clinical model construction

Variables in the training group underwent univariate logistic regression analysis and multivariate logistic regression analysis to identify the independent predictors significantly linked to the Ki-67, thus constructing a clinical model. For each predictor, odds ratios (OR) were calculated as measures of relative risk, along with 95% confidence intervals (CI).

### Tumour segmentation and radiomics feature extraction

Three-dimensional (3D) segmentation of regions of interest (ROI) was conducted using ITK-SNAP software (Version 3.8, www.itksnap.org). With careful exclusion of adjacent renal parenchyma and perinephric fat, the contours were meticulously drawn within the tumour boundaries on NP and CMP images.

To standardize the CT images before feature extraction, procedures such as image resampling and gray-level discretization were applied. A total of 3376 radiomics features were extracted from CMP and NP images using Pyradiomics software. These features were categorized into four groups: (1) intensity statistic features: This group consisted of 18 features designed to quantitatively describe the distribution of voxel intensities within the ROIs, utilizing commonly used and basic metrics. (2) shape features: Comprising 14 3D features, this group reflected the shape and size characteristics of the ROIs. (3) texture features: This group comprised 93 features calculated by gray level dependence matrix (GLDM), gray level co-occurrence matrix (GLCM), gray level run length matrix (GLRLM), gray level size zone matrix (GLSZM), and neighboring gray tone difference matrix (NGTDM), which quantified the heterogeneity differences of ROIs. (4) filter and wavelet features: This group included the intensity and texture features derived from wavelet and filter transformations of the original images. These transformations were achieved by applying filters such as square, square root, exponential, logarithm, gradient, lbp_two dimensional (2D), lbp_3D_k, and wavelet (wavelet-LLL, wavelet-LLH, wavelet-LHL, wavelet-LHH, wavelet-HLL, wavelet-HLH, wavelet-HHL and wavelet-HHH). Detailed instructions regarding the radiomics features can be found in the Pyradiomics document (Version 3.0) available at https://pyradiomics.readthedocs.io.

To assess inter- and intra-observer reproducibility of ROI contouring, 50 randomly selected CT data were evaluated by two radiologists. After an interval of 1 month, Reader 1 repeated the ROI segmentations, allowing for an evaluation of intra-observer reproducibility. The assessment of feature extraction agreement was conducted by evaluating the intra- and inter-class correlation coefficients (ICCs). An ICC value greater than 0.75 was deemed as indicative of satisfactory inter- and intra-observer reproducibility. The remaining segmentations were conducted by Reader 1.

### Construction of the radiomics signatures

In order to mitigate the risk of overfitting the signature, we employed a three-step process to reduce the dimensionality of the features. Initially, radiomics features exhibiting intra- and inter-reader ICCs exceeding 0.75 were preserved, thus guaranteeing the mitigation of potential subjectivity in the delineation of ROIs. Secondly, we employed the maximum relevance minimum redundancy (mRMR) method to select the 30 most relevant radiomics features for prediction while eliminating irrelevant and redundant ones. Finally, the least absolute shrinkage and selection operator algorithm (LASSO) was utilized to identify the optimal radiomics features. Subsequently, these selected features were amalgamated to constitute the radiomics signature (RS), following which a radiomics score (Rad-score) was calculated for each individual patient.

### Radiomics nomogram construction and performance assessment of different models

Based on the analysis of the clinical information, these independent predictors, in addition to the radiomic features, were utilized to construct a radiomics nomogram. To evaluate the discriminative performance of the clinical model, RS, and radiomics nomogram, the area under the curve (AUC) of the receiver operating characteristics (ROC) curve and Delong test was calculated for both the training and validation groups. Additionally, sensitivity, specificity, and accuracy metrics were computed for the three models. In order to assess the clinical utility of the three models when applied to the validation set, decision curve analysis (DCA) was conducted. This involved quantifying the net benefits at various threshold probabilities.

### Follow-up and survival analysis

The final follow-up date was July 31, 2019. The endpoint of this study was the recurrence-free survival (RFS), determined by measuring the duration from the date of surgery to the occurrence of either a recurrence, the last recorded negative follow-up, or patient demise. The median follow-up was 50 months (range: 1-118 months). After surgery, patients received regular follow-up assessments at intervals of every 6 to 12 months during the initial 2 years, followed by annual evaluations. Follow-up data, including physical exams and images, were collected from medical records. Additionally, telephone enquiries and medical insurance records were utilized to gather relevant information.

Survival curves based on the pathological Ki-67 expression status and Ki-67 expression status identified by three models were generated using the Kaplan-Meier method, and their concordance index (C-index) was calculated. The C-index serves as a measure of the proportion of correctly ordered pairs of individuals with predicted survival times [27], and its computation relies on Harrell’s C statistics [28]. A C-index score of approximately 0.70 is indicative of a well-performing model, while a score around 0.50 suggests random background. A higher C-index indicates a more accurate prognostic prediction [29].

### Statistical analysis

The statistical analysis was conducted using SPSS software (Version 26.0). Continuous variables were assessed through either independent t-tests or Mann-Whitney U tests, while categorical variables were analyzed using the chi-square test or Fisher’s exact test, as appropriate. Univariate and multivariate logistic regression analysis, mRMR, ICC, LASSO Cox regression, survival analysis, AUC, C-index, and DCA were carried out using R statistical software (Version 3.3.3, https://www.r-project.org). Statistical significance was determined based on a two-sided *P*-value < 0.05.

## Result

### Clinical model construction

The characteristics of the patients in the training and validation groups are detailed in Table 2. In the training group, lumbago, ECOG-PS, PLT and tumour size showed significant differences between the high Ki-67 expression group and low Ki-67 expression group. After multiple logistic regression analysis, only tumour size (*p* < 0.05, odds ratio = 1.024, 95%CI, 1.007 to 1.042) remained an independent predictor in the clinical model (Table 3).

**Table 2:** Characteristics of ccRCC patients in the high and low Ki-67 expression groups

Variables	Training group (*n* = 130)				Validation group (*n* = 55)		
	High expression (*n* = 34)	Low expression (*n* = 96)	*P*-value		High expression (*n* = 14)	Low expression (*n* = 41)	*P*-value
Age (years)	58.26 ± 8.26	55.01 ± 11.14	0.122		58.00 ± 9.54	55.93 ± 11.35	0.543
Gender			0.397				0.957
Female	10	36			4	14	
Male	24	60			10	27	
Hematuria			0.161				0.808
Absent	25	81			12	34	
Present	9	15			2	7	
Lumbago			0.023*				0.889
Absent	22	80			10	32	
Present	12	16			4	9	
ECOG-PS			0.031*				0.485
0	14	60			6	22	
1–2	20	36			8	19	
Hemoglobin			0.062				0.046*
Absent	21	75			7	32	
Present	13	21			7	9	
Leukocyte count			0.623				0.978
Absent	33	89			13	36	
Present	1	7			1	5	
PLT			0.037*				0.186
Absent	25	85			11	39	
Present	9	11			3	2	
Calcium			1				1
Absent	33	95			14	41	
Present	1	1			0	0	
Necrosis			0.508				0.566
Absent	30	90			12	39	
Present	4	6			2	2	
Tumour size (mm)	69.71 ± 26.42	49.43 ± 25.01	< 0.001*		72.07 ± 36.03	51.17 ± 27.55	0.028*
ALP (U/L)	77.00	69.00	0.157		66.00	72.70	0.288
Creatinine (µmol/L)	85.19 ± 15.79	80.60 ± 20.88	0.246		79.37 ± 17.23	77.03 ± 17.55	0.668
BUN (mmol/L)	5.17 ± 1.31(5.22)	5.57 ± 1.51(5.44)	0.171 (0.234)		5.15 ± 1.28	5.41 ± 1.37	0.533

***ECOG-PS***, Eastern Cooperative Oncology Group Performance Status, ***PLT***, platelet, ***ALP***, alkaline phosphatase, ***BUN***, Blood urea nitroge

**Table 3:** Univariate and multivariate logistic regression analysis of the preoperative clinical of training group

Variables	Univariate logistic regression			Multivariate logistic regression	
	OR (95% CI)	*P*-value		OR (95% CI)	*P*-value
Age	1.031 (0.992–1.071)	0.124			
Gender	1.440 (0.618–3.354)	0.398			
Hematuria	1.944 (0.759–4.978)	0.166			
Lumbago	2.727 (1.126–6.607)	0.026*		2.814 (0.723–10.951)	0.136
ECOG-PS	2.381 (1.072–5.290)	0.033*		0.711 (0.170–2.980)	0.641
Hemoglobin	2.211 (0.951–5.142)	0.065			
Leukocyte count	0.385 (0.046–3.252)	0.381			
PLT	2.782 (1.036–7.467)	0.042*		1.515 (0.397–5.785)	0.544
Calcium	2.879 (0.175–47.339)	0.459			
Necrosis	2.000 (0.528–7.569)	0.307			
Tumour size	1.029 (1.013–1.045)	< 0.001*		1.028 (1.006–1.050)	0.011*
ALP	1.004 (0.995–1.013)	0.390			
Creatinine	1.012 (0.992–1.032)	0.245			
BUN	0.817 (0.611–1.092)	0.172			

***ECOG-PS***, Eastern Cooperative Oncology Group Performance Status, ***PLT***, platelet, ***ALP***, alkaline phosphatase, ***BUN***, Blood urea nitrogen

### Radiomics signature construction

**Fig. 2:**
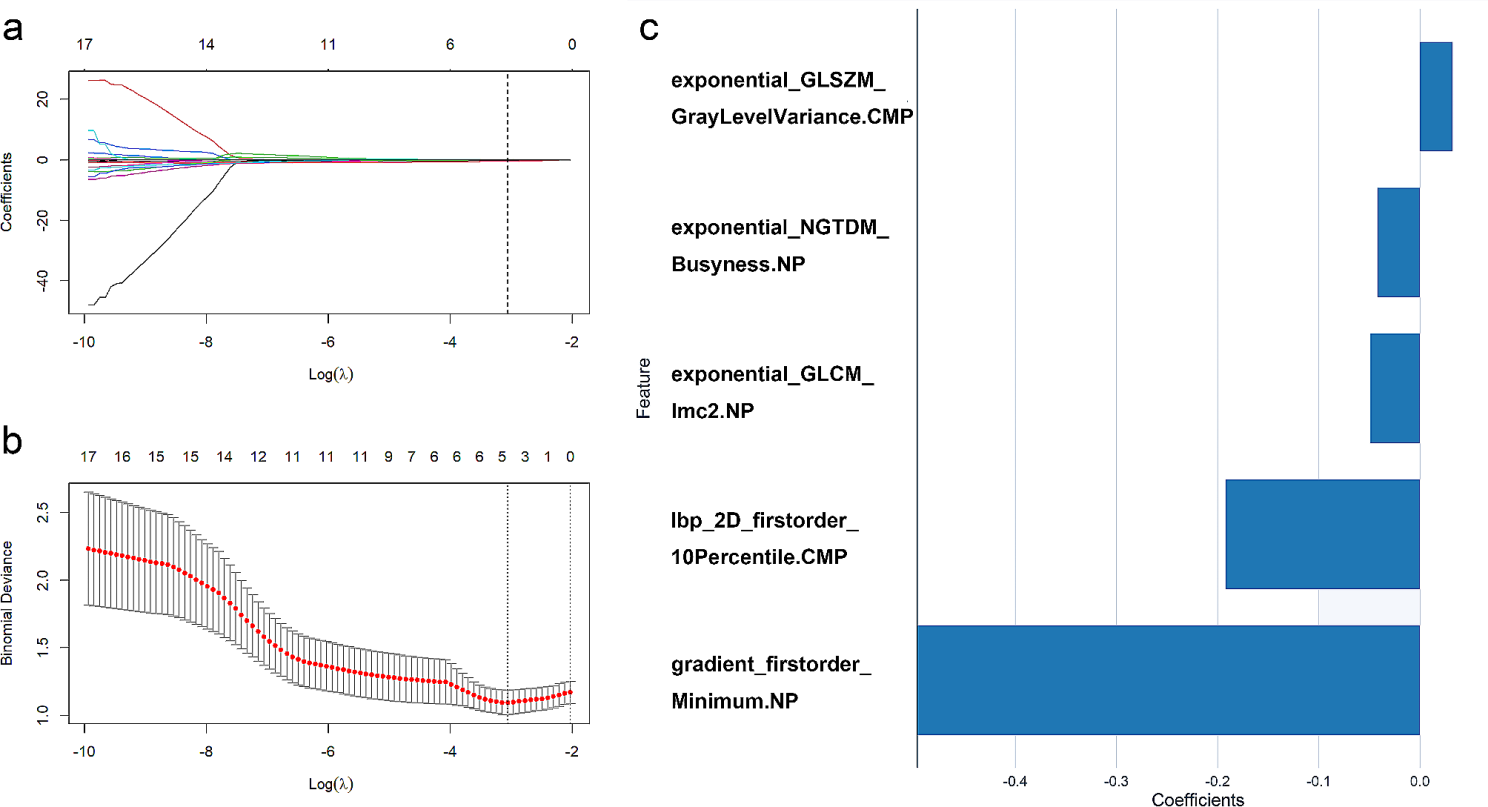
Steps for radiomics feature downscaling and selection. **a** LASSO path map, tuning parameter (λ) selection; **b** A coefficient profile plot, versus the selected log λ value; **c** 5 radiomics features with non-zero coefficients were selected; *LASSO* the least absolute shrinkage and selection operator algorithm

Among the 3376 radiomics features, 2530 demonstrated favorable inter- and intra-observer agreement. 30 most relevant radiomics features were selected using mRMR and subsequently employed into the LASSO regression model to identify the most valuable features. Ultimately, the RS was constructed based on 5 selected features (Table 4), and their correlation coefficients are presented in Fig. 2. The Rad-score was calculated using the following formula:

**Table 4:** Selected radiomics features

Feature		Meaning
lbp_2D_firstorder_10Percentile.CMP		This feature is derived from LBP texture analysis and represents a first-order statistical measure based on the 10th percentile value of pixel intensity distribution within an image or region of interest. It provides information about the lower range of gray levels present in the image, which can be useful for detecting subtle changes associated with pathology.
gradient_firstorder_Minimum.NP		The minimum gradient magnitude computed over all directions is represented by this feature. Gradients capture edge information, and their magnitudes indicate how abrupt these edges are across different scales. The minimum value taken among various gradients helps identify regions where the structure is less pronounced.
exponential_ NGTDM _Busyness.NP		Busyness refers to a measure of activity or complexity within an image patch. In the context of NGTDM features, it could reflect the amount of variation observed between neighboring pixels when considering multiple gray level differences simultaneously.
exponential_ GLSZM _GrayLevelVariance.CMP		GLSZM quantify co-occurrences of zones with similar sizes but varying intensities; GrayLevelVariance specifically describes how much individual grayscale values vary inside these zones relative to their mean value. This feature captures heterogeneity within structures under observation.
exponential_ GLCM _Imc2.NP		GLCM records the frequency of occurrence of pixel intensity pairs at different positions and directions. Imc2 was a specific element or submatrix within a GLCM. Such elements encode relationships between pairs of pixels at certain distances and orientations, providing insights into spatial arrangement and regularity/irregularity of tissues under study.

***CMP***, corticomedullary phase, ***NP***, nephrographic phase, ***LBP***, Local Binary Patterns, ***NGTDM***, Neighborhood Gray Tone Difference Matrix, ***GLSZM***, Grayscale zone matrices, ***GLCM***, Gray-level co-occurrence matrix

Rad-score = - (0.192*lbp_2D_firstorder_10Percentile.CMP)

+ (0.032*exponential_ GLSZM _GrayLevelVariance.CMP).

− (0.042*exponential_ NGTDM _Busyness.NP).

− (0.497*gradient_firstorder_Minimum.NP).

− (0.049*exponential_ GLCM _Imc2.NP) -1.117.

The Rad-score of the high Ki-67 expression group demonstrated a significant increase in comparison to the low Ki-67 expression group in the training (*p* < 0.001, 95%CI, -1.479 to -0.722) and validation groups, respectively (*p* < 0.001, 95%CI, -1.484 to -0.515).

### Radiomics nomogram construction

**Fig. 3:**
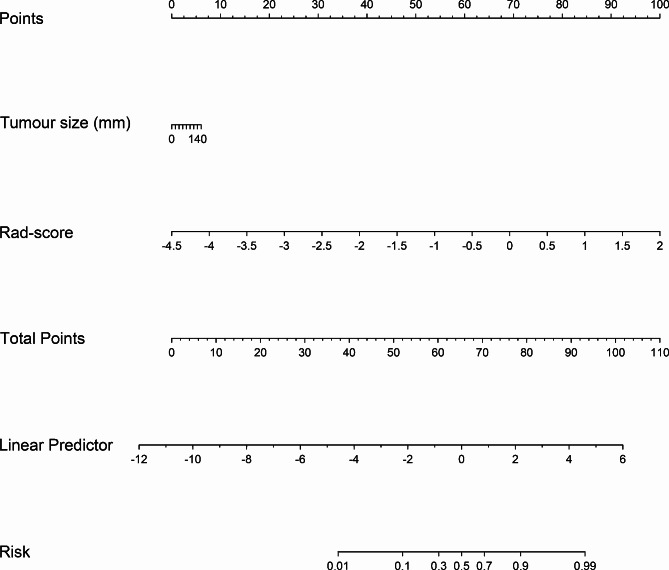
Radiomics nomogram based on radiomics features and the independent clinical predictor

As stated above, only tumour size emerged as an independent predictor of high-expression of Ki-67 (Table 3). Consequently, a radiomics nomogram, which integrates the radiomic features with the independent clinical predictor (tumour size), was formulated (Fig. 3). Notably, Hosmer-Lemeshow test showed good calibration in the training group (*p* = 0.290) and the validation group (*p* = 0.214), indicating good performance of the nomogram in the evaluation of Ki-67 expression status.

### The discrimination performance of the different models

**Fig. 4:**
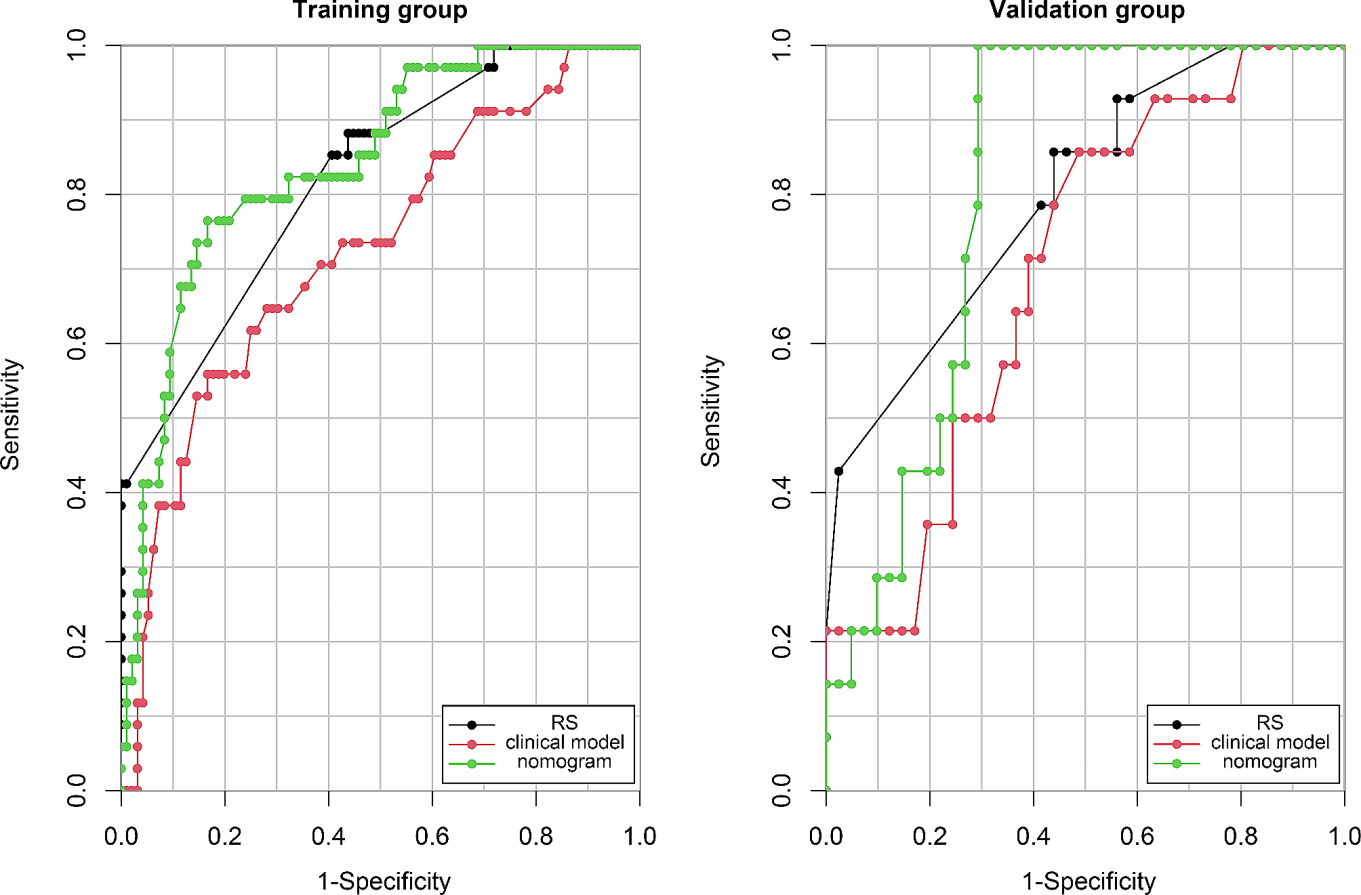
Receiver operating characteristic curves for each model in both the training (**a**) and validation **(b)** groups

Table 5 presents the diagnostic performance of the three CT models. Fig. 4 displays the ROC curves for each model in both the training and validation groups. The radiomics nomogram showed a higher AUC value than both clinical model and RS, and the Delong test demonstrated a superior performance for radiomics nomogram when compared to clinical model (*p* < 0.05). There was no significant difference in performance when compared to RS (*p* > 0.05).

**Table 5:** Diagnostic performance of the various models

Models	Training group (*n* = 130)					Validation group (*n* = 55)			
	AUC (95% CI)	Sensitivity	Specificity	Accuracy		AUC (95% CI)	Sensitivity	Specificity	Accuracy
Clinical model	0.724 (0.621–0.827)	0.559	0.833	0.762		0.698 (0.546–0.849)	0.5	0.756	0.691
RS	0.821 (0.745–0.897)	0.853	0.594	0.662		0.799 (0.668–0.929)	0.857	0.561	0.636
Nomogram	0.841 (0.765–0.917)	0.765	0.833	0.815		0.814 (0.704–0.925)	0.571	0.756	0.709

***RS***, radiomics signature, ***CI***, confidence interval

**Fig. 5:**
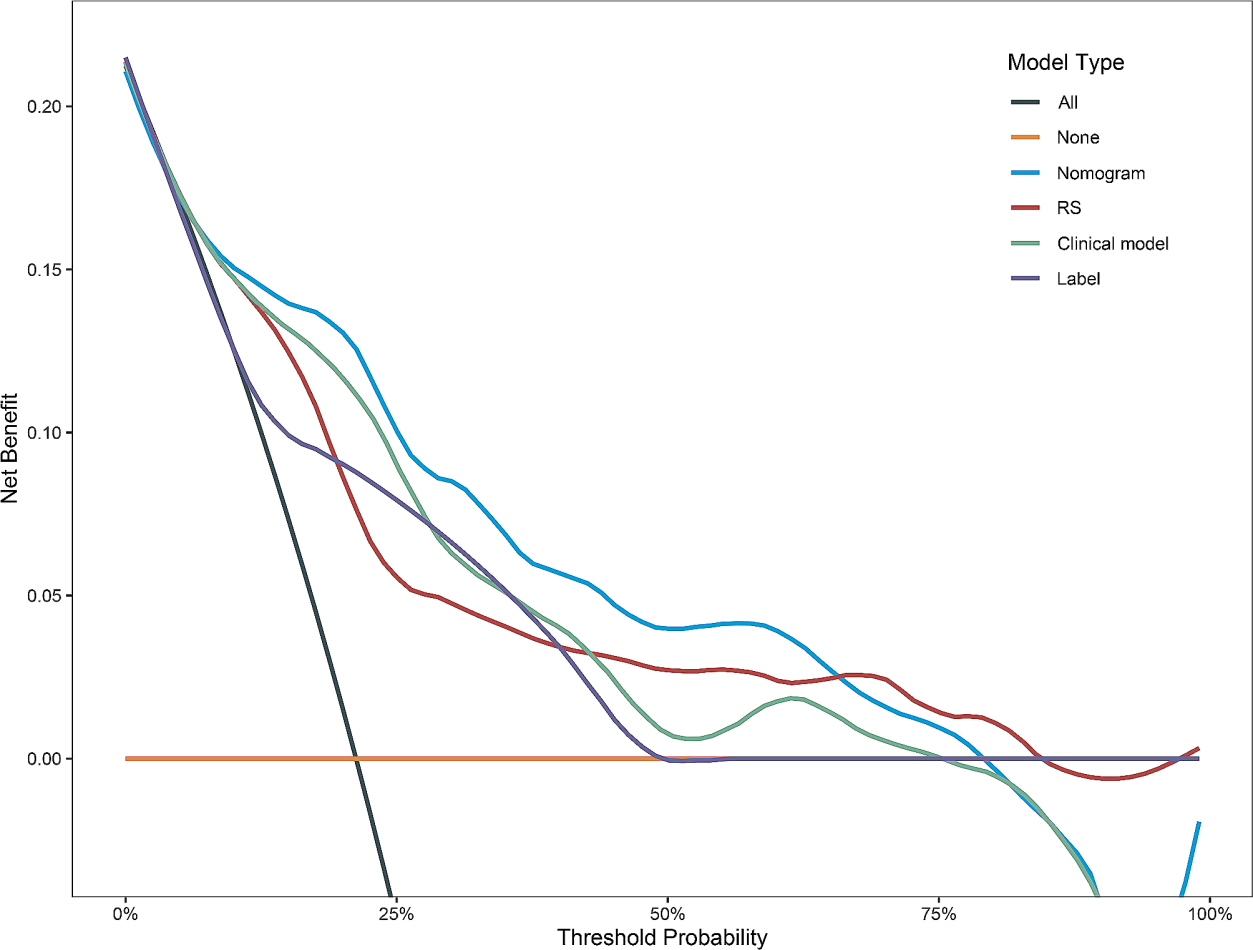
Decision curve analysis for the pathological Ki-67 expression status (**Label**,** purple line**) and three models. The radiomics nomogram (**Nomogram, blue line**) had better clinical net benefit than other models within a significant portion of the reasonable threshold probabilities for stratifying the Ki-67 index

In terms of the DCA results, the radiomics nomogram consistently exhibited a superior overall clinical net benefit compared to the other models within a significant portion of the reasonable threshold probabilities for stratifying the Ki-67 index. (Fig. 5).

### Survival prediction

**Fig. 6:**
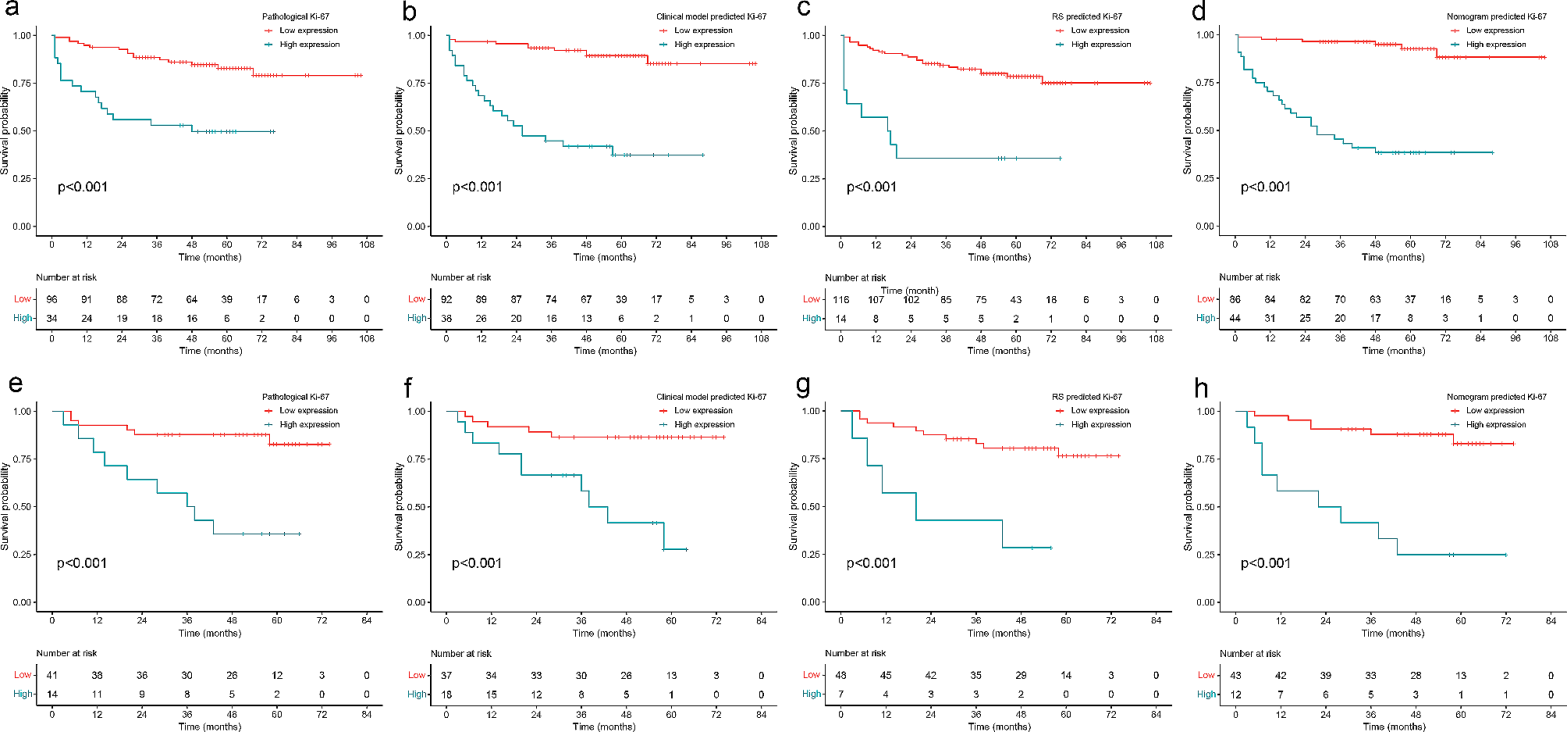
Survival curves of the pathological Ki-67 expression status, clinical model, RS and radiomics nomogram in training group **(a-d)** and validation group **(e-h)**, respectively

Figure 6 displays the survival curves based on the pathological Ki-67 expression status and Ki-67 expression status identified by three models, while Table 6 presents the corresponding C-index for both the training and validation groups. All models and the Ki-67 expression status demonstrated favorable outcomes (C-index > 0.7). The radiomics nomogram exhibited the highest C-index among all models, achieving 0.841in the training group and 0.820 in the validation group.

**Table 6:** The corresponding C-index for both the training and validation groups

Models	C-index (95% CI)		
	Training group (*n* = 130)		Validation group (*n* = 55)
Label	0.831 (0.728–0.933)		0.805 (0.716–0.883)
Clinical model	0.802 (0.727–0.877)		0.773 (0.666–0.881)
RS	0.819 (0.751–0.886)		0.794 (0.657–0.930)
Nomogram	0.841 (0.764–0.918)		0.820 (0.715–0.925)

***Label***, pathological Ki-67 expression status, ***RS***, radiomics signature, ***CI***, confidence interval

## Discussion

In our study, a radiomics nomogram integrating CT radiomic features with clinical parameters was developed and validated, which demonstrated great performance in effectively predicting the status of Ki-67 expression and the prognosis in ccRCC patients.

The Ki-67 nucleoprotein, a crucial biomarker associated with tumour heterogeneity and aggressiveness, demonstrates a significant correlation with the prognosis of various tumours [9, 12, 30, 31]. Based on previous research [13, 14, 32–34], it has proved as an independent predictor of malignant potential and a dependable prognostic tool for predicting outcomes in patients with ccRCC. In this study, we retrospectively utilized medical data from 185 ccRCC patients to analyze traditional clinical factors (age, gender, hematuria, lumbago, ECOG-PS, hemoglobin, leukocyte count, PLT, LDH, ALP, calcium, creatinine, BUN, tumour size and necrosis). Among these factors, tumour size was found to be the only significantly independent variable for predicting Ki-67 index stratification. However, tumour size, as an independent predictor, achieved a relatively lower AUC values of 0.724 and 0.698 in the training and validation groups, respectively, suggesting that the factor derived from conventional clinical data made a restricted contribution to the prediction of the Ki-67 expression status in ccRCC patients.

The significance of standardized preprocessing of CT images prior to feature extraction in diminishing variations stemming from diverse scanners and imaging parameters has been well-established [35]. In our study, the standardized preprocessing was adhered before feature extraction, including gray-level discretization and voxel size resampling. This approach enabled us to obtain features in line with the feature definitions prescribed by the Image Biomarker Standardization Initiative (IBSI) [36]. By doing so, we minimized potential confounding effects and the discrepancies arising from differences in image specifications to ensure improved reproducibility of CT features [35]. However, future investigations should consider exploring the complex interplay between these imaging parameters and radiomics performance. Such efforts could lead to the development of robust normalization methods or adaptive feature sets that can accommodate varying imaging protocols without compromising accuracy, aiming towards establishing universal benchmarks for CT screening programs worldwide.

Radiomics has been proven to facilitate the precise assessment of tumour heterogeneity, which has been shown to have an important prognostic relationship in various malignancies [37], including lung cancer [38], breast cancer [39], esophageal cancer [40], hepatocellular carcinoma [41], and RCC [42]. Our group has also found a definitive correlation between the heterogeneity based on CT radiomics and the clinical outcomes of patients with ccRCC. The rad-score of the high Ki-67 expression group, composed of the most valuable 5 radiomics features, demonstrated a significant elevation compared to that of the low Ki-67 expression group, indicating greater pixel differences between the images and increased tumour heterogeneity [43]. Besides, radiomics has also been shown to have great potential to predict high Ki-67 expression in malignancies (Table 7). Zhang et al. developed a CT-based radiomics nomogram (AUC = 0.784) to predict high Ki-67 expression in gastrointestinal stromal tumours [26]. Wu et al. constructed a radiomics nomogram based on a combination of radiomic features and clinical factors (AFP and Edmondson grades) to predict Ki-67 expression status in hepatocellular carcinoma, they found that the efficacy of the combined nomogram (AUC = 0.819) in predicting Ki-67 expression status was significantly better than the clinical models (AUC = 0.630, 0.699) [44]. Similarly, a radiomics nomogram combining the radiomic features with independent clinical factors was constructed by our group, achieving the highest predictive performance (AUC: training, 0.884; validation, 0.819) and good calibration capability (*p* > 0.05 in the Hosmer-Lemeshow test). The Delong test results demonstrated better performance in predicting than clinical factors (*p* < 0.05) and comparable performance to RS (*p* > 0.05), highlighting the important role of radiomics in predicting Ki-67 expression status. Furthermore, DCA provided additional support for the enhanced clinical utility of the radiomics nomogram when contrasted with the RS. This suggests that clinical factors serve a complementary function, and their integration offers superior practicality in a clinical context. These findings suggest that the radiomics nomogram holds promise as a dependable clinical diagnostic tool for stratifying the Ki-67 index, which can contribute to precise clinical decision-making.

**Table 7:** Studies based on radiomics to predict Ki-67 expression status and the prognosis of ccRCC

	Radiomics predict Ki-67 expression status			Radiomics predict the prognosis of ccRCC	
Author	Zhang et al. [26].	Wu et al. [44].		Gao et al. [45].	He et al. [46].
Year	2020	2022		2021	2022
Disease Type	gastrointestinal stromal tumors	hepatocellular carcinoma		ccRCC	ccRCC
No. of Patients	339	172		214	493
Method^*^	The radiomic nomogram including the radiomic signature and tumor size	The radiomics nomogram based on radiomic signature and clinical factors		The prognostic nomogram containing radiomic signature and clinicopathological parameters	The radiomics nomogram combining radiomics with clinical risk factors
AUC (95%CI)^**^	0.784 (0.701–0.868)	0.819 (0.688–0.912)		0.768 (NA)	12 months: 0.826 (0.717–0.936) 36 months: 0.805 (0.694–0.916) 60 months: 0.760 (0.635–0.886)

Note: * Take the best model; ** Take the validation set AUC firstly, otherwise take the training set AUC

***ccRCC***, clear cell renal cell carcinoma, ***AUC***, area under curve, ***CI***, confidence interval

Previous studies have shown that radiomics also has significant potential for predicting the prognosis of ccRCC (Table 7). Gao et al. established a prognostic nomogram (AUC = 0.768) containing a radiomic signature and clinicopathological parameters to predict the outcomes in ccRCC [45]. While He et al. developed a radiomics score-based nomogram to predict prognosis and the AUC of predictive performance were 0.826 at 12 months, 0.805 at 36 months, and 0.76 at 60 months [46]. Our study evaluated the predictive efficacy of models for RFS in ccRCC by using the Ki-67 index as a stratification factor. The pathological Ki-67 expression status as well as the Ki-67 expression status predicted by the three models were found to be associated with RFS of RCC. The radiomics nomogram, combining radiomic features and clinical features, showed the highest predictive performance among the models in the training group (C-index: 0.841; 95%CI, 0.764 to 0.918) and the validation group (C-index: 0.820; 95%CI: 0.715 to 0.925). Patients with high Ki-67 expression predicted using the radiomics nomogram are at high risk of recurrence and should be given active follow-up and adjuvant therapy. Conversely, patients with low risk can be followed up routinely using.

However, certain limitations need to be acknowledged in this study. First, despite meticulously applied rigorous inclusion and exclusion criteria, retrospective studies pose inherent challenges in eliminating selection bias. Second, manual segmentation for defining tumor regions introduces the potential for error and variance. Future research should explore semi-automatic methods for improved accuracy. Third, the relatively small sample size of 185 patients limits the study’s predictive power. Finally, although CT images undergo standardized preprocessing, the use of different scanners with variable characteristics can impact the radiomics score.

Despite the progress made in the application of radiomics in ccRCC, several challenges remain to be addressed. First, most studies have relatively small sample sizes and lack large-scale prospective validation. Second, differences in radiomic feature extraction and selection methods across studies limit the reproducibility and generalizability of the results. In the future, standardized radiomics analysis need to be established to improve the robustness and interpretability of radiomics signatures. Moreover, integrating radiomics with multi-omics data, such as clinical characteristics, histopathological features, and genomic profiles, may further enhance the predictive performance of radiomics signatures. We believe that with the continuous advancement of artificial intelligence techniques and the accumulation of big data, radiomics has the potential to play a greater role in personalized diagnosis and treatment.

In summary, we have developed and verified a radiomics nomogram that combines radiomic features with clinical factors. This nomogram demonstrates strong predictive performance for assessing Ki-67 expression status and exhibits a great capacity for prognostic prediction in patients with ccRCC, providing a valuable resource for tailoring personalized treatment strategies and facilitating comprehensive clinical monitoring.

## Conclusions

Our study demonstrated that the radiomics nomogram was the superior predictive model of Ki-67 expression status and prognosis compared with clinical model or RS alone. The effect of Ki-67 expression status on prognosis was also verified. Radiomics nomogram based on CT radiomics features and clinical factors may offer significant benefits in customizing individualized treatment approaches and enhancing thorough clinical surveillance for patients with ccRCC.

### Electronic supplementary material

Below is the link to the electronic supplementary material.

Supplementary Material 1

## Data Availability

The datasets used and/or analyzed during the current study are available from the corresponding author on reasonable request.

## Abbreviations

RCC: Renal cell carcinoma
ccRCC: Clear cell renal cell carcinoma
CMP: Corticomedullary phase
NP: Nephrographic phase
ECOG-PS: Eastern Cooperative Oncology Group Performance Status
PLT: Platelet count
LDH: Lactate dehydrogenase
ALP: Alkaline phosphatase
BUN: Blood urea nitrogen
OR: Odds ratios
CI: Confidence intervals
3D: Three-dimensional
2D: Two-dimensional
ROI: Regions of interest
GLDM: Gray level dependence matrix
GLCM: Gray level co-occurrence matrix
GLRLM: Gray level run length matrix
GLSZM: Gray level size zone matrix
NGTDM: Neighboring gray tone difference matrix
ICC: Intra- and inter-class correlation coefficients
mRMR: Maximum relevance minimum redundancy
LASSO: Least absolute shrinkage and selection operator algorithm
RS: Radiomics signature
Rad-score: Radiomics score
AUC: Area under the curve
ROC: Receiver operating characteristics
DCA: Decision curve analysis
RFS: Recurrence-free survival
C-index: Concordance index
IBSI: Image Biomarker Standardization Initiative
