# Supplementary material for: Radiomics nomogram based on CT radiomics features and clinical factors for prediction of Ki-67 expression and prognosis in clear cell renal cell carcinoma: a two-center study
Source: Cancer Imaging. 2024 Aug 6;24:103. doi: 10.1186/s40644-024-00744-1 (PMC11302839; doi:10.1186/s40644-024-00744-1)
Supplement: Supplementary file 1 — Supplementary Material 1 [file 40644_2024_744_MOESM1_ESM.pdf]

SPRINGER NATURE

Change of authorship request form - Journals (pre-acceptance)

Important information. Please read.

- This form should be used by authors to request any change in authorship (adding/deleting authors) including changes in corresponding authors. This form should not be used for name changes. Please fully complete all sections. Use black ink and block capitals and provide each author's full name with the given name first followed by the family name.
- By signing this declaration, all authors guarantee that the order of the authors are in accordance with their scientific contribution, if applicable as different conventions apply per discipline, and that only authors have been added who made a meaningful contribution to the work.
- Please note, in author collaborations where there is formal agreement for representing the collaboration, it is sufficient for the representative or legal guarantor (usually the corresponding author) to complete and sign the Authorship Change Form on behalf of all authors, **next to the added/removed author(s)**. **(Complete Section 3, followed by Section 6.)**
  In author collaborations where there is no formal agreement for representing the collaboration and **there are more than 10 authors**, one may sign for all, provided the signer appends correspondence that attests that each of the authors have agreed to the change **and the added/removed authors sign the form**. **(Complete Section 3, followed by Section 6.)**
- Please note, we cannot investigate or mediate any authorship disputes. If you are unable to obtain agreement from all authors (including those who you wish to be removed) you must refer the matter to your institution(s) for investigation. Please inform us if you need to do this.
- If you are not able to return a fully completed form within **30 days** of the date that it was sent to the author requesting the change, we may have to withdraw your manuscript. We cannot publish manuscripts where authorship has not been agreed by all authors (including those who have been removed).
- Incomplete forms will be rejected.
- Please return/upload this form, fully completed, to the Journals Editorial Office. The Journal and/or Publisher will consider the information you have provided to decide whether to approve the proposed change in authorship. We may decide to contact your institution for more information or undertake a further investigation, if appropriate, before making a final decision.

SPRINGER NATURE

Change of authorship request form - Journals (pre-acceptance)

Section 1: Please provide the current title of manuscript

Manuscript ID no.: CAIG-D-24-00022

Title: Radiomics nomogram based on CT radiomics features and clinical factors for prediction of Ki-67 expression and prognosis in clear cell renal cell carcinoma: a two-center study

Section 2: Please provide the previous authorship, in the order shown on the manuscript before the changes were introduced. Please indicate the corresponding author by adding (CA) behind the name.

	First name(s)	Family name	ORCID or SCOPUS id, if available
1st author	BEN	LI	0009-0006-7523-0144
2nd author	JIE	ZHU	
3rd author	ZHAISONG	GAO	
4th author	HAILEI	SHI	
5th author	PEI	NIE	
6th author	JU	ZHANG	
7th author	YUAN	ZHUANG	
8th author	ZHENGUANG	WANG (CA)	
9th author	GUANGJIE	YANG (CA)	0000-0003-1783-4282
10th author			

Please use an additional sheet if there are more than 10 authors.

SPRINGER NATURE Springer Nature is one of the world's leading global research, educational and professional publishers, created in May 2015 through the combination of Nature Publishing Group, Palgrave Macmillan, Macmillan Education and Springer Science+Business Media.

SPRINGER NATURE Change of authorship request form - Journals (pre-acceptance)

Section 3: Please provide a justification for change. Please use this section to explain your reasons for changing the authorship of your manuscript, e.g. what necessitated the change in authorship? Please refer to the (journal) policy pages for more information about authorship. Please explain why omitted authors were not originally included and/or why authors were removed on the submitted manuscript.

Dear Editor,
Greetings!
After an in-depth review and discussion of our manuscript, our team has decided to reassess the contributions of the authors. In this process, we have recognized the significant contributions made by Yanmei Wang and Yuchao Xu in the areas of deep learning and statistical processing. With the unanimous agreement of all authors, we have decided to list Yanmei Wang as the third author and Yuchao Xu as the fourth author of the article. We are fully aware that this change may impose additional work on the journal's publication process, for which we express our sincere apologies. We promise to cooperate fully with the journal to complete all necessary updates. Please refer to the title page for the updated author order. We believe that this adjustment will more accurately reflect the contributions of each author to this research work and ensure the transparency and academic integrity of the article. We look forward to your understanding and support, and hope that this change will not adversely affect our review process.
Thank you for your time and consideration.
Sincerely,
Guangjie Yang

Section 4: Proposed new authorship. Please provide your new authorship list in the order you would like it to appear on the manuscript. Please indicate the corresponding author by adding (CA) behind the name. If the Corresponding Author has changed, please indicate the reason under section 3.

	First name(s)	Family name (this name will appear in full on the final publication and will be searchable in various abstract and indexing databases)	Affiliated institute	E-mail address
1st author	BEN	LI	Department of Nuclear Medicine, The Affiliated Hospital of Qingdao University, China.	2229896647@qq.com
2nd author	JIE	ZHU	Department of Scientific Research Management and Foreign Affairs, The Affiliated Hospital of Qingdao University, China.	zhujieok1990@163.com
3rd author	YANMEI	WANG	GE Healthcare China, Pudong New Town, Shanghai, China.	wangyanmei_cams@163.com
4th author	YUCHAO	XU	School of Nuclear Science and Technology, University of South China, China.	yxu40@hotmail.com
5th author	ZHAISONG	GAO	Department of Nuclear Medicine, The Affiliated Hospital of Qingdao University, China	Gaozhaisong@126.com
6th author	HAILEI	SHI	Department of Pathology, The Affiliated Hospital of Qingdao University, China	18661801331@163.com
7th author	PEI	NIE	Department of Radiology, The Affiliated Hospital of Qingdao University, China	niepei@qdu.edu.cn
8th author	JU	ZHANG	Department of Nuclear Medicine, The Affiliated Hospital of Qingdao University, China	zj_1823@qq.com

Please use an additional sheet if there are more than 10 authors.

SPRINGER NATURE Springer Nature is one of the world's leading global research, educational and professional publishers, created in May 2015 through the combination of Nature Publishing Group, Palgrave Macmillan, Macmillan Education and Springer Science+Business Media.

SPRINGER NATURE

Change of authorship request form - Journals (pre-acceptance)

Section 4: Additional sheet

	First name(s)	Family name (this name will appear in full on the final publication and will be searchable in various abstract and indexing databases)	Affiliated institute	E-mail address
9th author	YUAN	ZHUANG	Department of Nuclear Medicine, The Affiliated Hospital of Qingdao University, China.	1944036722@qq.com
10th author	ZHENGUANG	WANG (CA)	Department of Nuclear Medicine, The Affiliated Hospital of Qingdao University, China.	wangzhenguang@qdu.edu.cn
11st author	GUANGJIE	YANG (CA)	Department of Nuclear Medicine, The Affiliated Hospital of Qingdao University, China.	ygj_2815@qdu.edu.cn

SPRINGER NATURE Springer Nature is one of the world's leading global research, educational and professional publishers, created in May 2015 through the combination of Nature Publishing Group, Palgrave Macmillan, Macmillan Education and Springer Science+Business Media.

SPRINGER NATURE # Change of authorship request form - Journals (pre-acceptance)

Section 5: Author contribution, Acknowledgement and Disclosures. Please use this section to provide a new disclosure statement and, if appropriate, acknowledge any contributors who have been removed as authors and ensure you state what contribution any new authors made (if applicable per the journal or book (series) policy). Please ensure these are updated in your manuscript - <u>after approval of the change(s)</u> - as our production department will not transfer the information in this form to your manuscript.

New acknowledgements:

Not applicable.

New Disclosures (financial and non-financial interests, funding):

Not applicable.

New Author Contributions statement (if applicable per the journal policy):

Ben Li and Guangjie Yang: designed the study;
Yanmei Wang, Yuchao Xu and Ben Li: analyzed the data;
Ben Li, Jie Zhu, Hailei Shi, Ju Zhang, and Yuan Zhuang: conducted the experiments;
Jie Zhu, Pei Nie and Zhenguang Wang: advised study and revised the draft;
Ben Li: wrote the draft.
All authors read and approved the final manuscript.

State 'Not applicable' if there are no new authors.

SPRINGER NATURE Springer Nature is one of the world's leading global research, educational and professional publishers, created in May 2015 through the combination of Nature Publishing Group, Palgrave Macmillan, Macmillan Education and Springer Science+Business Media.

SPRINGER NATURE

Change of authorship request form - Journals (pre-acceptance)

Section 6: Declaration of agreement. *All* authors, unchanged, new and removed *must* sign this declaration.

(NB: Please print the form, (docu-)sign and return/upload a scanned copy. Please note that signatures that have been inserted as an image file are acceptable as long as it is handwritten.
T[illegible] *** Please delete as appropriate. Delete all of the bold if you were on the original authorship list and are remaining as an author.**

	First name	Family name		Signature	Date
1st author	BEN	LI	I agree to the proposed new authorship shown in section 4 /and the proposed change in corresponding author	BEN LI	2024. 3. 27
2nd author	JIE	ZHU	I agree to the proposed new authorship shown in section 4 /and the proposed change in corresponding author	JIE ZHU	2024. 3. 27
3rd author	YANMEI	WANG	I agree to the proposed new authorship shown in section 4 **/and the addition /and** the proposed change in corresponding author	YANMEI WANG.	2024. 3. 27
4th authors	YUCHAO	XU	I agree to the proposed new authorship shown in section 4 **/and the addition /and** the proposed change in corresponding author	YUCHAO XU	2024. 3. 27
5th author	ZHAISONG	GAO	I agree to the proposed new authorship shown in section 4 /and the proposed change in corresponding author	ZHAISONG GAO	2024. 3. 27
6th author	HAILEI	SHI	I agree to the proposed new authorship shown in section 4 /and the proposed change in corresponding author	HAI LEI SHI	2024. 3. 27
7th author	PEI	NIE	I agree to the proposed new authorship shown in section 4 /and the proposed change in corresponding author	PEI NIE	2024. 3. 27

SPRINGER NATURE Springer Nature is one of the world's leading global research, educational and professional publishers, created in May 2015 through the combination of Nature Publishing Group, Palgrave Macmillan, Macmillan Education and Springer Science+Business Media.

Change of authorship request form - Journals (pre-acceptance)

	First name	Family name		Signature	Date
8th author	JU	ZHANG	I agree to the proposed new authorship shown in section 4 /and the proposed change in corresponding author	JU ZHANG	2024.3.27
9th author	YUAN	ZHUANG	I agree to the proposed new authorship shown in section 4 /and the proposed change in corresponding author	YUAN ZHUANG	2024.3.27
10th author	ZHENGUANG	WANG	I agree to the proposed new authorship shown in section 4 /and the proposed change in corresponding author	ZHENGUANG WANG	2024.3.27

Please use an additional sheet if there are more than 10 authors.

Section 6: Additional sheet

	First name	Family name		Signature	Date
11st author	GUANGJIE	YANG	I agree to the proposed new authorship shown in section 4 /and the proposed change in corresponding author	GUANGJIE YANG	2024.3.27
12nd author			I agree to the proposed new authorship shown in section 4 /and the proposed change in corresponding author		
13rd author			I agree to the proposed new authorship shown in section 4 /and the proposed change in corresponding author		

SPRINGER NATURE Springer Nature is one of the world's leading global research, educational and professional publishers, created in May 2015 through the combination of Nature Publishing Group, Palgrave Macmillan, Macmillan Education and Springer Science+Business Media.

In case of author collaborations with formal agreement:

	Name of consortium/consortia	First name	Family name		Signature	Date
Representative/legal guarantor				I agree to the proposed new authorship shown in section 4 /**and the addition/removal*of my name to the authorship list** /and the proposed change in corresponding author		

Both added/removed authors should complete the information in the first table under Section 6.

---- End of form ----
